# Role of Necroptosis and Immune Infiltration in Human Stanford Type A Aortic Dissection: Novel Insights from Bioinformatics Analyses

**DOI:** 10.1155/2022/6184802

**Published:** 2022-04-16

**Authors:** Fuqiang Liu, Tao Wei, Lin Liu, Fangxia Hou, Cuixiang Xu, Hua Guo, Wei Zhang, Meijuan Ma, Yulian Zhang, Qi Yu, Junkui Wang

**Affiliations:** ^1^Department of Cardiology, Shaanxi Provincial People's Hospital, Xi'an, China; ^2^Shaanxi Key Laboratory of Ischemic Cardiovascular Diseases & Institute of Basic and Translational Medicine, Xi'an Medical University, Xi'an, China; ^3^Shaanxi Key Laboratory of Integrative Traditional Chinese and Western Medicine for Prevention and Treatment of Cardiovascular Diseases, Xianyang, China; ^4^Department of Cardiovascular Surgery, Shaanxi Provincial People's Hospital, Xi'an, China; ^5^Shaanxi Provincial Key Laboratory of Infection and Immune Diseases, Shaanxi Provincial People's Hospital, Xi'an, China; ^6^Department of Nursing, Shaanxi Provincial People's Hospital, Xi'an, China

## Abstract

**Background:**

Stanford type A aortic dissection (TAAD) is one of the most life-threatening cardiovascular emergencies with high mortality and morbidity, and necroptosis is a newly identified type of programmed cell death and contributes to the pathogenesis of various cardiovascular diseases. However, the role of necroptosis in TAAD has not been elucidated. This study was aimed at determining the role of necroptosis in TAAD using bioinformatics analyses.

**Methods:**

The RNA sequencing dataset GSE153434 and the microarray dataset GSE52093 were obtained from Gene Expression Omnibus (GEO) database. Differentially expressed genes of necroptosis (NRDEGs) were identified based on differentially expressed genes (DEGs) and necroptosis gene set. Gene set enrichment analysis (GSEA) was applied to evaluate the gene enrichment signaling pathway in TAAD. The STRING database and Cytoscape software were used to establish and visualize protein-protein interaction (PPI) networks and identify the key functional modules of NRDEGs. Gene Ontology (GO) and Kyoto Encyclopedia of Genes and Genomes (KEGG) pathway enrichment analyses of NRDEGs were also performed. Additionally, Spearman correlations were used to construct the necroptosis-related transcription factor-target genes regulatory network, immune infiltration patterns were analyzed using the ImmuCellAI algorithm, and the correlation between immune cell-type abundance and NRDEGs expression was investigated. The expression levels of NRDEGs and immune infiltration were additionally verified in the GSE52093 dataset.

**Results:**

We found that the necroptosis pathway was considerably enriched and activated in TAAD samples. Overall, 25 NRDEGs were identified including MLKL, RIPK1, and FADD, and among them, 18 were verified in the validation set. Moreover, GO and KEGG enrichment analyses found that NRDEGs were primarily involved in the tumor necrosis factor signaling pathway, nucleotide-binding oligomerization domain-like receptor signaling pathway, and interleukin-17 signaling pathway. The imbalance of Th17/Treg cells was identified in the TAAD samples. Furthermore, correlation analysis indicated that expression of NRDEGs was positively associated with proinflammatory immune-cell infiltrations and negatively associated with anti-inflammatory or regulatory immune-cell infiltrations.

**Conclusions:**

The present findings suggest that necroptosis phenomenon exists in TAAD and correlates with immune cell infiltration, which indicate necroptosis may promote the development of TAAD through activating immune infiltration and immune response. This study paves a new road to future investigation of the pathogenic mechanisms and therapeutic strategies for TAAD.

## 1. Introduction

Aortic dissection (AD) is one of the most common catastrophic cardiovascular diseases caused by tearing of the intima with consecutive bleeding into the media of the aortic wall [1, 2]. According to the Stanford classification, type A aortic dissections (TAAD) involve the presence of dissection proximal to the left subclavian artery, while type B aortic dissections are limited to the descending aorta without any proximal extension. The incidence of AD was approximately 3 to 6 per 100,000 people per year and was rising dramatically over the last decade [3, 4]. Acute AD followed by aortic rupture results in a high mortality rate of up to 90% in the absence of prompt intervention. Indeed, aortic rupture is an uncommon but potentially life-threatening condition that often results in death without prompt treatment. Thus, understanding the etiology and pathogenesis of AD can guide the clinical diagnosis and therapies, thereby improving clinical outcomes.

Immune infiltration has been the hot spot in the field of cancer in the recent decade, and a small but growing number of studies have shown that the immune-inflammatory pathway is involved in pathophysiological and metabolic alterations of AD [5, 6]. Notably, an immune infiltrate has been found within the middle and outer tunics of dissected aortic specimens [7]. The recall and activation of macrophages inside the middle tunic have been considered an early alteration of AD, which leads to matrix degradation and neoangiogenesis [8]. Moreover, several bioinformatic comprehensive analyses also confirm the difference of immune infiltrate between AD and normal control [9, 10]. However, the mechanisms underlying immune infiltration in AD remain poorly understood.

Necroptosis is a newly identified form of programmed cell death with hallmark features of necrosis [11]. Necroptosis integrates some features of necrosis and apoptosis, including loss of membrane integrity, organelle swelling, cell lysis, leakage of intracellular components, and initiation by death receptor ligands [11, 12]. The classical necroptosis pathway is described as follows [ [12]]: The death receptor ligands, such as tumor necrosis factor (TNF) *α* and interferon-*γ*, trigger death receptors and recruit receptor-interacting protein kinases 1 (RIPK1) and 3 (RIPK3) to compose a regulatory necrosome complex. Activated RIPK3 binds and activates the downstream executor mixed lineage kinase domain-like protein (MLKL) by phosphorylation at the serine 232 site. Phosphorylated MLKL shifts into an oligomerized state that facilitates the formation of membrane-disrupting pores, finally leading to necrotic death. Due to the rapid plasma membrane rupture, proinflammatory intracellular contents are released and subsequently induce an inflammatory response. Recently, accumulating studies have investigated necroptosis mechanisms involved in the pathogenesis of cardiovascular diseases, including aortic aneurysm and dissection [13–17]. For example, studies found that the expression of RIPK3 was significantly enhanced in elastase-induced or angiotensin II-challenged apolipoprotein E ^−/−^ mouse models of acute aortic aneurysm or dissection, and inhibition of RIPK3-mediated necroptosis could prevent aortic enlargement and acute aortic aneurysm or dissection formation. The findings indicate a vital role of necroptosis for necroptosis in the formation of aneurysms and dissection [18–20]. However, the phenomenon has not yet been empirically confirmed in human samples.

In this study, we conducted a systematic bioinformatics analysis using the Gene Expression Omnibus (GEO) database to outline the immune infiltration landscape and determine whether and how necroptosis contributes to the development of TAAD. Moreover, we explored the potential functional mechanism and key genes of necroptosis in TAAD. Additionally, we deliberated the relationship between necroptosis and infiltrating immune cells to gain a better understanding of the potential molecular immunity process during the development of TAAD.

## 2. Materials and Methods

### 2.1. Data Acquisition

**Figure 1:**
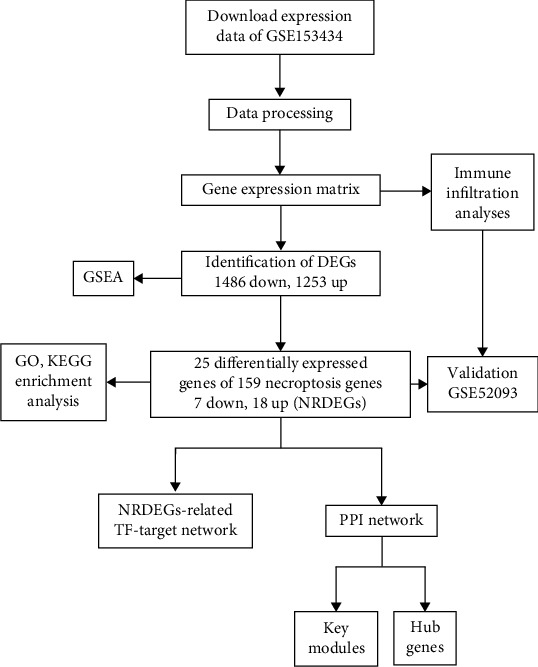
Flowchart of the multistep screening strategy on bioinformatics data.

The RNA-seq transcriptome data of patients with TAAD and the corresponding clinical data were obtained from GEO by R package “GEO query.” GSE153434 dataset (FPKM values) contains 10 AD ascending aorta tissues and 10 normal aortic tissues, which was performed on the GPL20795 (Illumina HiSeq X Ten Homo sapiens) platform [21]. The data were normalized using the RMA (Robust multiarray analysis) algorithm implemented in the limma R-package. The microarray dataset GSE52093 served as a validation dataset used for independently external validation, which included data from 7 AD ascending aorta tissues and 5 normal aortic tissues. All data used in the study were obtained from the GEO, and hence, ethics approval and informed consent were not required. The workflow chart is shown in Figure 1.

### 2.2. Selection of Necroptosis-Related Genes

We screened differentially expressed genes (DEGs) using the R language package “limma (v 3.48.3),” and the cutoff was adjusted *p* < 0.05 [22]. Volcano plots and heatmaps of DEGs were generated using the ggplot2 R package (https://ggplot2.tidyverse.org) and the heatmap R package (https://CRAN.R-project.org/package=pheatmap). Eight necroptosis genes in the gene set M24779.gmt were retrieved from the Gene Set Enrichment Analysis (GSEA) (http://www.gsea-msigdb.org/gsea/index.jsp). Additionally, we collected the profiles of 159 genes related to necroptosis from the Kyoto Encyclopedia of Genes and Genomes (KEGG) Pathway databases (https://www.genome.jp/dbget-bin/www_bget?pathway+hsa04217). Supplemental material S1 provides detailed genes information. We identified differentially expressed genes of necroptosis (NRDEGs) via the Venn diagram package (1.7.1).

### 2.3. Protein-Protein Interaction (PPI) Network and Identification of Hub Genes and Key Modules

The PPI network of NRDEGs was visualized by the STRING database (https://string-db.org/) and Cytoscape software (version 3.8.2) [23, 24]. CytoHubba and Molecular Complex Detection (MCODE) of Cytoscape software were used to identify hub genes and key modules of the PPI network [25, 26].

### 2.4. Functional Enrichment Analysis

To investigate potential biological functions between AD and normal control, Gene Ontology (GO) enrichment analysis and KEGG pathway analysis were conducted by R package clusterProfiler. Only terms with false discovery rate <0.05 were considered statistically significant enriched pathways. Moreover, the “clusterProfiler” package was used to perform GSEA for the potential mechanism of c2 (c2.cp.kegg.v7.5.1.entrez.gmt) and c5 (c5.bp.v7.5.1.entrez.gmt) in the molecular signature database (MSigDB) [27]. The number of random sample permutations was set to 1,000, and the significance threshold was set to *p* < 0.05. Gene set variation analysis (GSVA) was used to evaluate the association between biological pathways and gene signatures based on expression profile data. The gene set “c2.cp.kegg.v7.5.1” for GSVA analysis was downloaded from the MSigDB database.

### 2.5. Necroptosis-Related Transcription Factor- (TF-) Target Genes Regulatory Network

To investigate the molecular mechanism of necroptosis, the TRRUST, MSigDB, RegNetwork, and hTFtarget databases were utilized to identify TF-target gene interaction pairs and retrieve those involved in necroptosis regulation. The necroptosis-related transcription factor–target gene regulatory interaction network was generated, and the NRDEGs obtained from the GSE153434 dataset were used to construct the necroptosis-related TF-target regulatory network, which was calculated using Spearman correlations and visualized using Cytoscape (V 3.8.0). The lowest Spearman correlation coefficient of |0.6| demonstrates unequivocally that biologically significant gene correlations occur.

### 2.6. Immune Infiltration Analyses

A comprehensive prediction of immune cell abundance was provided by the Immune Cell Abundance Identifier (ImmuCellAI) (http://bioinfo.life.hust.edu.cn/web/ImmuCellAI) [28]. ImmuCellAI is an online tool that estimates the abundance of 24 immune cell types (including 18 T-cell subtypes and 6 other immune cells [B cells, natural killer cells, monocytes, macrophages, neutrophils, and dendritic cells]) using the gene expression data based on a gene set signature method. The infiltration score was defined as the sum of all percentages of 24 infiltrating immune cells. Wilcoxon rank-sum test and Spearman correlation were used to explore the correlation between the key regulators and the infiltration levels of immune cells and NRDEGs.

### 2.7. Validation of NRDEGs and Immune Infiltration in GSE52093

The expressions of the NRDEGs were extracted from GSE52093, and the difference between TAAD and normal samples was calculated and visualized by package ggpurb and ggplot2 respectively. A *p* value of < 0.05 was considered significant. Immune cell abundance in GSE52093 was also estimated using ImmuCellAI.

## 3. Results

### 3.1. Identification of DEGs and NRDEGs

**Figure 2:**
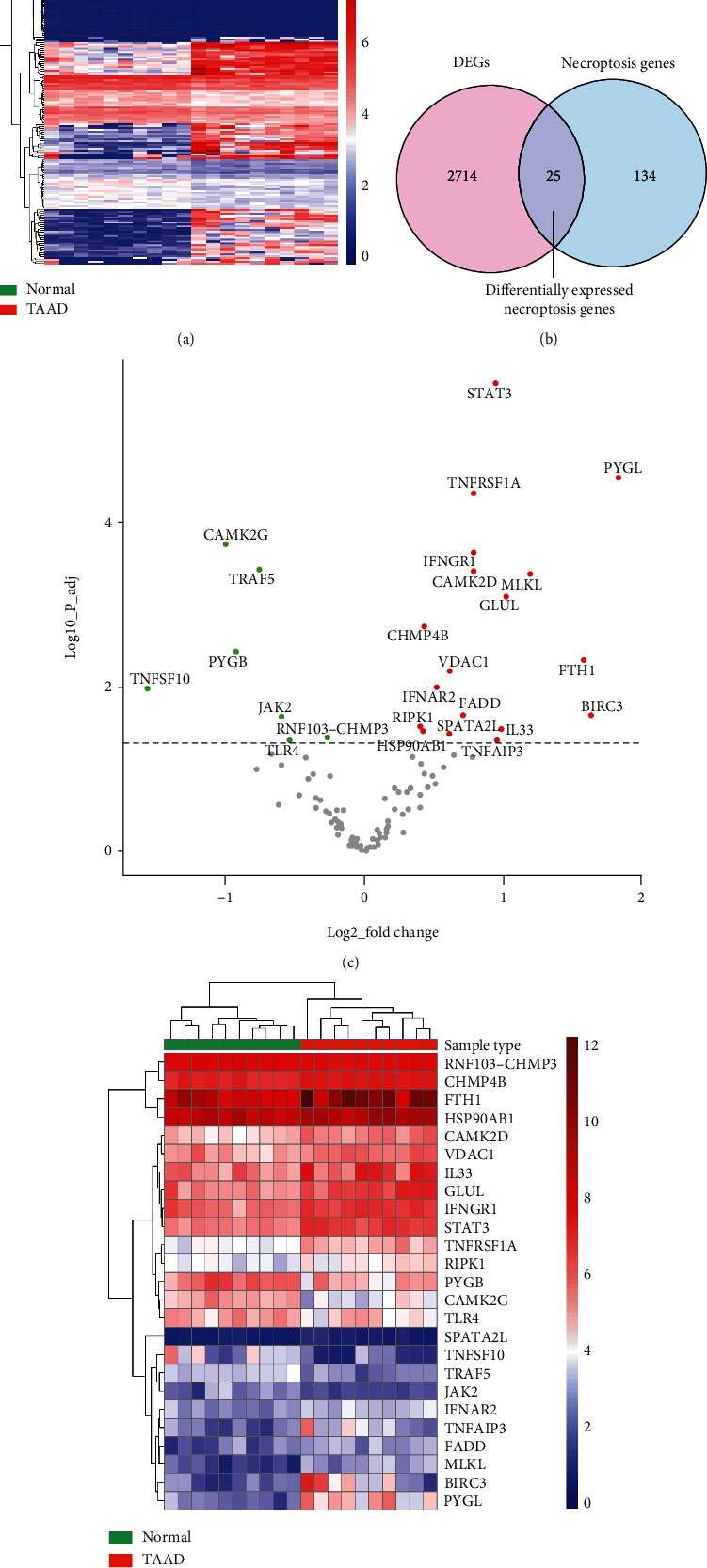
Screening of the differentially expressed necroptosis-related genes (NRDEGs) in Stanford type A aortic dissection. (a) Clustered heatmap of differentially expressed genes (DEGs) in GSE153434. (b) Venn diagram showing the overlap of genes between DEGs in GSE153434 and necroptosis-related genes in Kyoto Encyclopedia of Genes and Genomes pathway databases. (c) The volcano plot of NRDEGs. (d) Clustered heatmap of NRDEGs.

We revealed a total of 2739 DEGs between TAAD and normal samples in GSE153434, with 1253 genes upregulated and 1486 genes downregulated. Figure 1(a) depicts the expression patterns of the most differentially expressed genes clustered as a heatmap for visualization purposes (Figure 2(a)). We overlapped NRDEGs with the DEGs in GSE153434 and selected 25 overlapped NRDEGs for further analyses, of which 18 were upregulated and 7 were downregulated. The Venn diagram analysis revealed 25 overlapped genes as NRDEGs, including MLKL and RIPK1 (Figure 2(b) and Supplemental material S2). The expression heatmap and volcano plot of NRDEGs are presented in Figures 2(c) and 2(d).

### 3.2. Profile of Gene Set Enrichment Analysis

**Figure 3:**
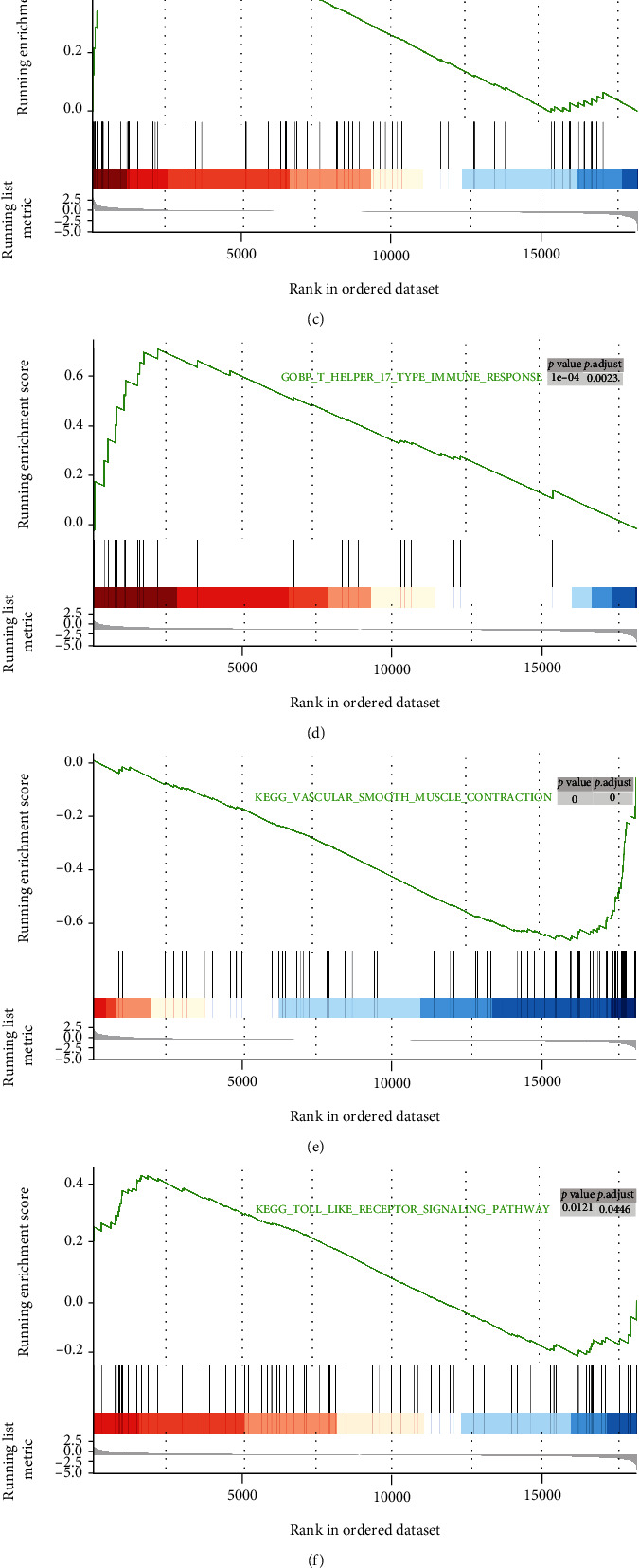
Enrichment plots from gene set enrichment analysis in Stanford type A aortic dissection.

GSEA was performed to identify predominant signaling pathways. Based on the enrichment analysis of pathways using GO and KEGG databases, 668 GO terms and 49 KEGG pathways were obtained (Supplemental material S3-S4). As shown in Figure 3(a), the necroptosis pathway was considerably enriched and predominantly upregulated (normalized enrichment score = 1.497) at a nominal *p* value of 0.005, indicating an overall strong link between necroptosis and TAAD. GSEA also revealed that gene expression relevant to apoptosis, nucleotide-binding and oligomerization domain- (NOD-) like receptor signaling pathway, TNF signaling pathway, Th17 cell differentiation, and Toll-like receptor signaling pathway was significantly enriched in AD (Figures 3(b)–3(g)).

### 3.3. PPI Network Analysis and Functional Modules' Construction

**Figure 4:**
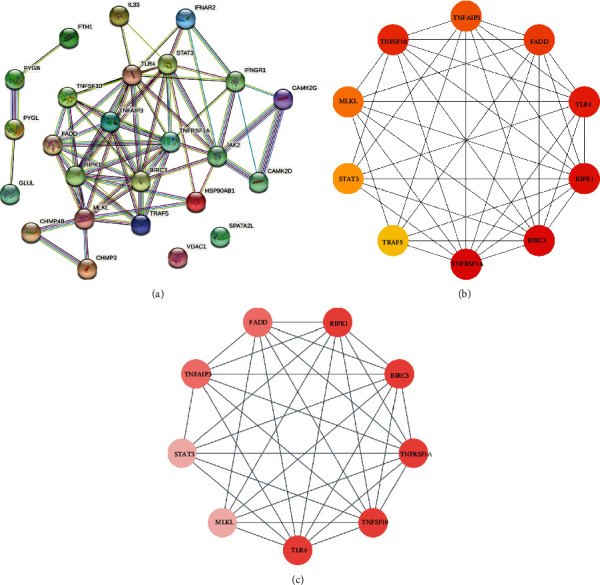
Protein-protein interaction (PPI) networks and key modules constructed by STRING and Cytoscape. (a) There are 69 edges and 25 nodes in the PPI network. (b) Top 10 hub genes explored by CytoHubba. (c) Molecular Complex Detection network clustering analysis.

PPI network of NRDEGs was constructed using the STRING database (Figure 4(a)), followed by analysis using Cytoscape software. The top 10 hub genes were identified based on the CytoHubba MCC algorithm by Cytoscape software (Figure 4(b)). These included TNFRSF1A, BIRC3, RIPK1, TLR4, FADD, TNFAIP3, TNFSF10, MLKL, STAT3, and TRAF5. The MCODE plugin was utilized to analyze important modules in the PPI network, and a module consisting of 9 nodes and 33 edges was recognized as a significant cluster in the PPI network. RIPK1, TLR4, and TNFSF10 occupied the center of the module (Figure 4(c)).

### 3.4. Functional Analyses and Mechanism Exploration

**Figure 5:**
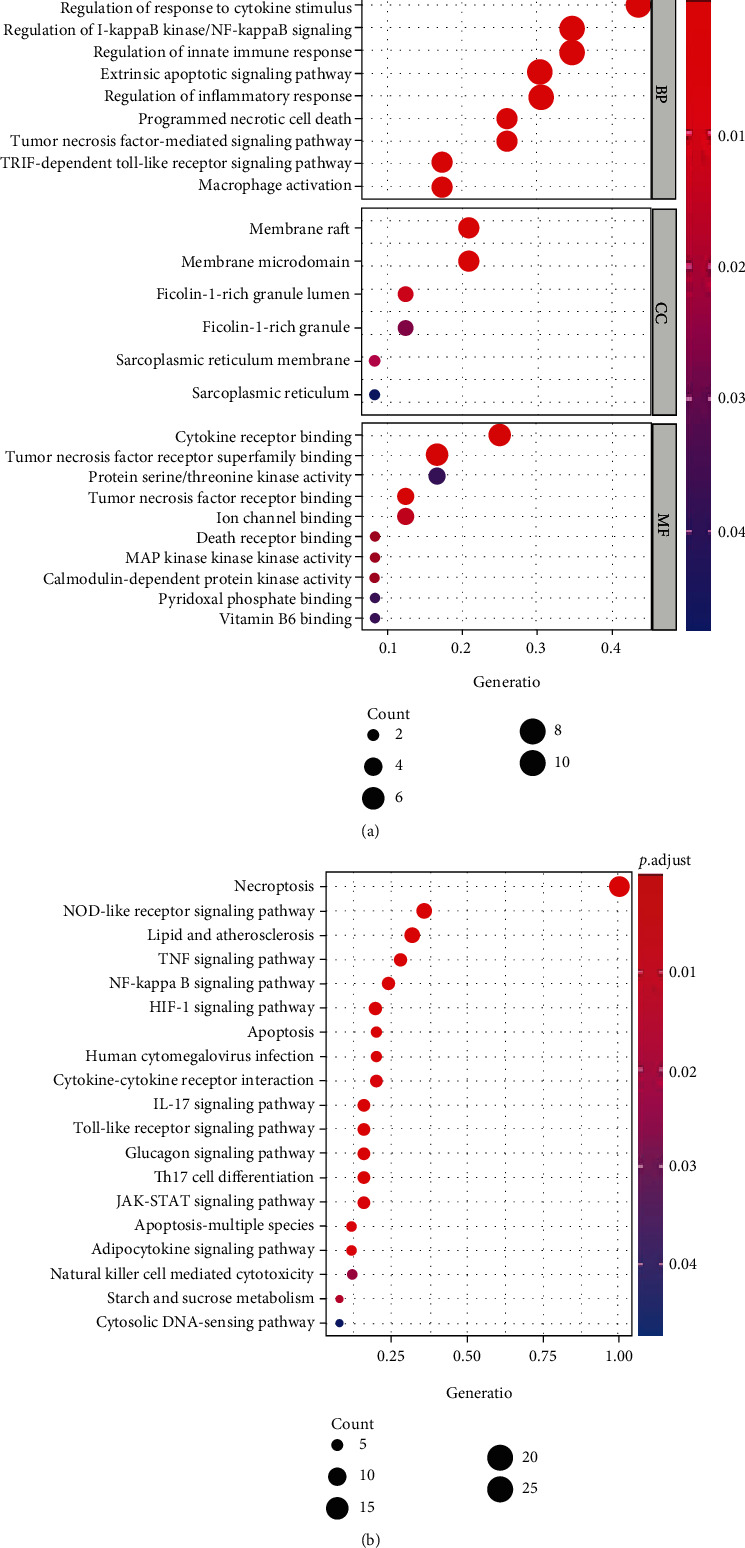
Enriched items in GO and KEGG analyses using necroptosis-related differentially expressed genes (NRDEGs). (a) Enriched items in GO analysis. (b) Enriched items in KEGG pathway analysis. GO: Gene Ontology; BP: biological process; CC: cellular component; MF: molecular function; KEGG: Kyoto Encyclopedia of Genes and Genomes.

We performed GO and KEGG enrichment analyses to determine the functions and related pathways of the NRDEGs. A total of 414 significantly related biological processes and 43 KEGG signaling pathways were obtained (Supplemental material S5). The GO enrichment analysis revealed enrichment of Toll-like receptor signaling pathway, innate immune response, TNF signaling pathway, apoptotic signaling pathway, response to cytokine stimulus, inflammatory response, and macrophage activation in biological processes. Moreover, we also demonstrated enrichment of ficolin-1-rich granule lumen, sarcoplasmic reticulum membrane, and membrane raft in cellular components. Furthermore, enrichment of TNF receptor binding, death receptor binding, and ion channel binding was also found in molecular function (Figure 5(a)). Most importantly, the KEGG analysis revealed that NRDEGs tended to be enriched in the following terms: necroptosis, apoptosis, NOD-like receptor signaling pathway, lipid and atherosclerosis, interleukin- (IL-) 17 signaling pathway, natural killer cell-mediated cytotoxicity, and cytokine-cytokine receptor interaction (Figure 5(b)).

### 3.5. Transcription Factor-Differentially Expressed Necroptosis-Related Genes Regulatory Network

**Figure 6:**
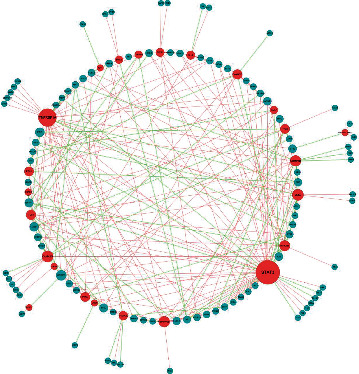
Transcription factor–necroptosis-related differentially expressed genes (NRDEGs) regulatory network in Stanford type A aortic dissection. The size of the dots reflects the degree, the red dots represent NRDEGs, and the blue dots represent transcription factors. The color of the edge lines in this network represents the correlation: bright red lines indicate a positive correlation, while light green lines indicate a negative correlation.

We predicted the regulatory relationship between transcription factors and NRDEGs through the Spearman correlation of expression levels and finally constructed a transcription factor-NRDEGs' network to highlight the critical processes driving the transcriptional control of NRDEGs. First, 2063 necroptosis-related TFs were downloaded from the TRRUST, MSigDB, RegNetwork, and hTFtarget databases and matched to NRDEGs. Second, by calculating the Spearman correlation coefficient of differently expressed mRNAs with a cutoff ∣cor | >0.6, interactions between transcription factors and NRDEGs were identified. This network contained 96 necroptosis-related TFs matched to 24NRDEGs and was displayed using Cytoscape 3.8.0 (Figure 6).

### 3.6. Relationships between the Necroptosis-Related Genes and Immune Infiltration

**Figure 7:**
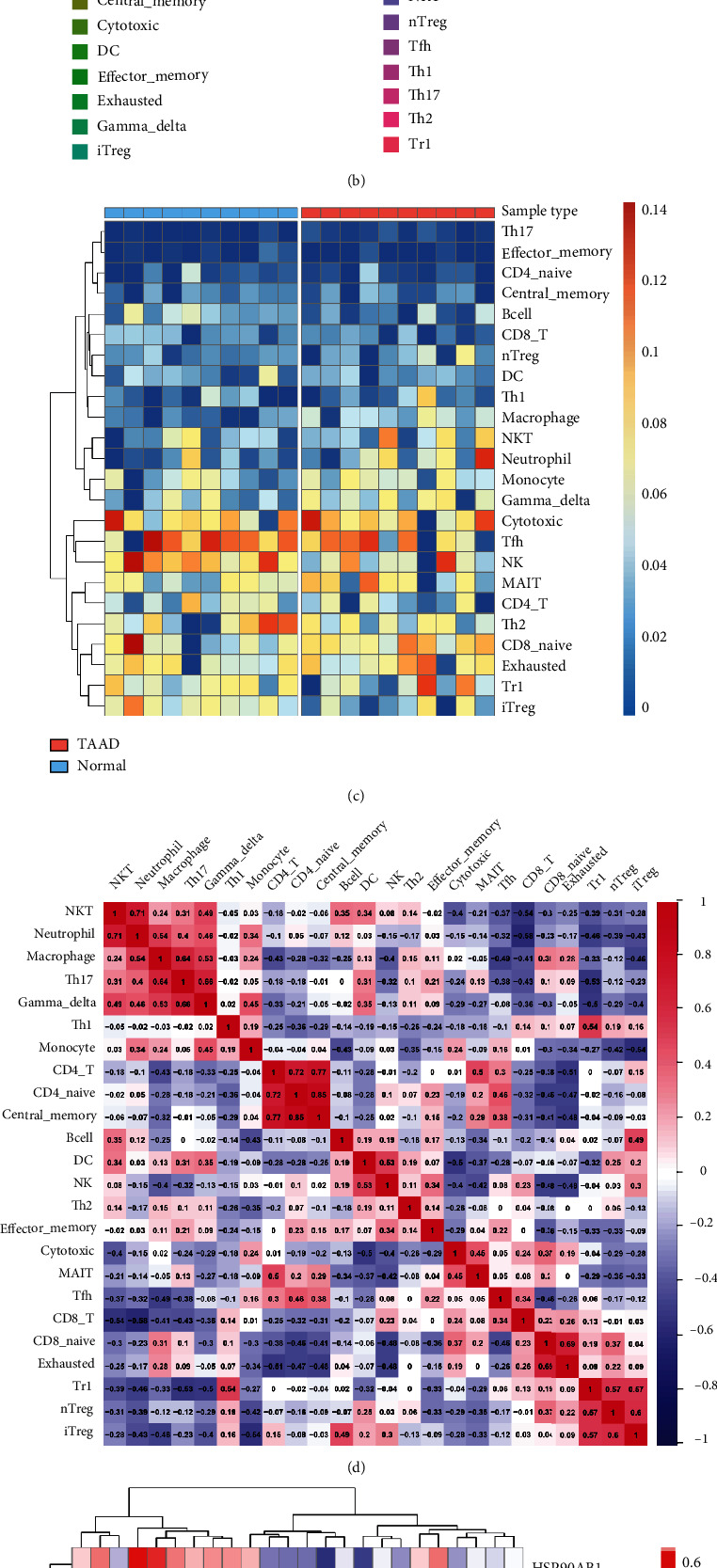
The landscape of immune infiltration in Stanford type A aortic dissection. (a) The violin plot of the immune cell proportions.(b) Stacked bar chart of the immune cell. (c) Heatmap of the proportions of 24 immune cell types. (d) The correlation matrix of immune cell proportions. (e) The correlation between differentially expressed necroptosis-related genes and immune cells.

In the enrichment analyses, we found that NRDEGs were highly enriched in immune-related pathways. We employed ImmuCellAI algorithms to evaluate the association between necroptosis and immune infiltration characterization in TAAD, and the results were promising. Among 24 immune cells, there were 6 cell types with significant differences in TAAD tissues compared with controls (Figures 7(a)–7(d)); among the 6 cell types, the scores of macrophages and Th17 and CD8 naive cells in the TAAD group were significantly enriched, whereas B cell, induced Treg, and natural killer cells were noticeably enriched in the normal group. Furthermore, we estimated the correlation efficiency between immune cells and NRDEGs to investigate their link and potential interaction. The NRDEGs were predominantly favorably correlated with macrophage and Th17 cell infiltrations and primarily negatively correlated with induced Treg cell infiltrations (Figure 7(e)).

### 3.7. Validation of NRDEGs and Immune Infiltration

**Figure 8:**
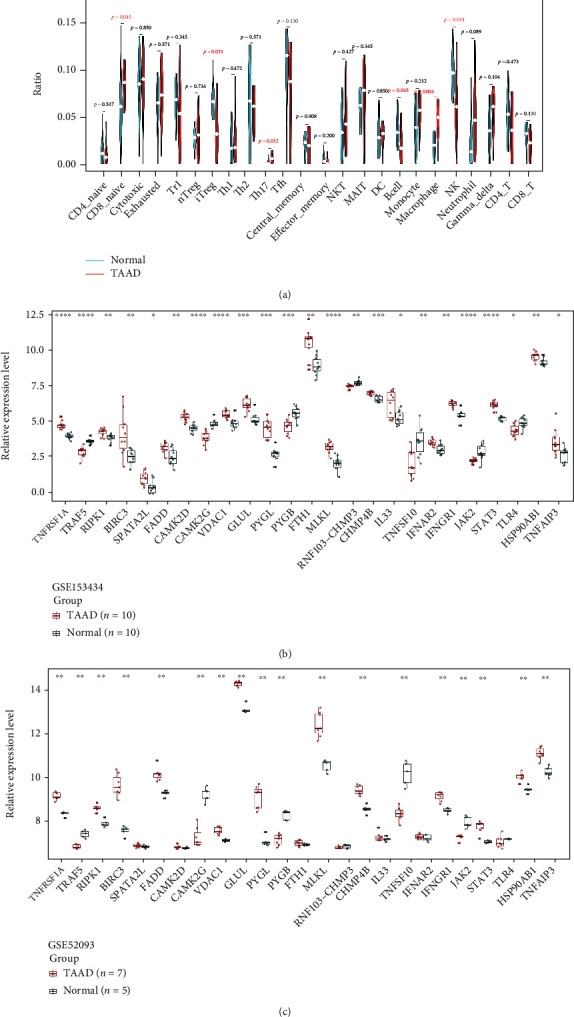
Validation of NRDEGs and immune infiltration (a) Expression of 25 NRDEGs in GSE151314. (b) Expression of 25 NRDEGs in GSE52093. (c) The violin plot of the immune cell proportions.

A dataset independent of the microarray dataset GSE52093 was used to validate the results. As described previously, the expression data of 25 NRDEGs were extracted and 18 NRDEGs were in accordance with the tendency in GSE153434, including TNFRSF1A, BIRC3, RIPK1, FADD, TNFAIP3, TNFSF10, MLKL, STAT3, and TRAF5 (Figures 8(a) and 8(b)). Immune cell infiltration was predominantly macrophages and Th17 and CD8 naive cells in the TAAD group, and induced Treg was enriched in the normal group (Figure 8(c)). In general, the results were relatively consistent between the training and validation set.

## 4. Discussion

Necroptosis plays a role in the pathophysiology of heart disease, including atherosclerosis, ischemia-reperfusion injury, and myocardial infarction. However, its role in TAAD remains vastly uncharacterized. Herein, we confirmed the existence of necroptosis in TAAD, as evidenced by (1) alterations in the expression of RIPK1, MLKL, and other 23 genes; (2) activation of necroptosis in GSEA; (3) effectual construction of the TF-NRDEGs regulatory network. Furthermore, the enrichment analysis revealed that NRDEGs were primarily engaged in immune-inflammatory response pathways, including TNF signaling pathway, NOD-like receptor signaling pathway, and IL-17 signaling pathway. Additionally, we observed a dramatic difference in immune cell content between TAAD and normal samples. Intriguingly, both RIPK1 and MLKL were positively associated with macrophages and Th17 infiltrations and negatively associated with Treg cells. To the best of our knowledge, this is the first bioinformatics report describing the coexistence of necroptosis in the pathogenesis of human TAAD.

The vascular smooth muscle cell (VSMC) loss is a prominent pathological characteristic of TAAD [6, 15, 29]. Progressive VSMC loss contributes to aortic dysfunction and degeneration, leading to aortic aneurysm, dissection, and ultimately rupture. Recently, emerging evidence has implicated the critical role of programmed cell death pathways in VSMC loss, including apoptosis, necroptosis, pyroptosis, and ferroptosis [15]. For example, studies have revealed activation of the intrinsic and extrinsic apoptosis pathways in VSMCs in aortic aneurysm and dissection [30, 31]. Moreover, NLR Family Pyrin Domain Containing 3- (NLRP3-) caspase-1 inflammasome could directly cleave and degrade contractile proteins in VSMCs, and NLRP3 or caspase-1 deficiency in mice significantly reduced angiotensin II-induced AD formation [32, 33], indicating that pyroptosis in VSMCs contributes to the development of TAAD. Additionally, although direct evidence regarding the role of ferroptosis in AD development is lacking, Zou et al. [34], using bioinformatic analysis, demonstrated differentially expressed ferroptosis-related genes in the normal and TAAD samples. Furthermore, much of the information about necroptosis and AD development has been derived from animal experiments [18, 19]. Notably, in the present study, we found that the apoptosis pathway was activated based on GSEA analysis, and importantly, the canonical necroptosis signaling pathway was identified in human TAAD samples. Therefore, various types of programmed cell death pathways may together contribute to the pathogenesis of AD development. The findings shed light on the development of new therapeutic strategies for preventing aortic disease progression.

In addition to necroptosis and apoptosis, GSEA also identified several other biologic pathways that were significantly and potentially relevant to TAAD. Notably, the TNF signaling pathway, which may promote extrinsic apoptosis and necroptosis, was enriched and activated in TAAD. TNF*α*-mediated and Toll-like receptor- (TLR-) induced necroptosis have been widely investigated [35]. TLR signaling pathway, which represents antimicrobial responses, was downregulated in TAAD; therefore, TNF*α* might be the primary factor regulating necroptosis. We also found that the vascular smooth muscle contraction pathway and muscle cell development pathway were inhibited, which partially explains the VSMC loss in TAAD. Intriguingly, multiple immune-related pathways were also enriched in AD samples, including B cell-mediated immunity, NOD-like receptor signaling pathway, Th17 cell differentiation, T-cell differentiation involved in immune response, and Th2 cell differentiation. These findings were further confirmed in subsequent immune infiltration analyses.

In the present study, we applied ImmuCellAI to estimate the abundance of 24 immune cells based on the microarray profiles of aortic tissues samples and obtained a comprehensive view of the immune infiltration landscape. The macrophage score was higher in TAAD; the finding is consistent with that in previous studies [36, 37]. Macrophages are the first immune cells that infiltrate aortic media and adventitia to promote a local inflammatory response after dissection, and they are also associated with some complications of TAAD [5, 38–40]. Indeed, macrophages have been demonstrated to induce vascular SMC apoptosis, elastic fiber breakdown, and neovascularization, resulting in deterioration and separation of the aorta wall [40, 41]. Moreover, T-cells are the most critical immune cells in cell-mediated immune responses, and various T-cell subsets play varied roles depending on the inflammatory event in the TAAD [8, 42, 43]. Our results revealed a higher abundance of Th17 and a lower proportion of Tregin TAAD. These results are consistent with data obtained in peripheral blood [44]. The imbalance of Th17/Treg cells has been reported to contribute to atherosclerosis and plaque rupture. Our study is the first to demonstrate the role of the imbalance of Th17/Treg cells in TAAD. Notably, the association between necroptosis and immune infiltration is complex. Necroptosis could impact immune cells in two fundamentally different ways. On the one hand, necroptosis is one of the forms of cell death in a variety of immune cells. For example, necroptosis of macrophages could drive necrotic core formation in atherosclerosis [16]. On the other hand, necroptotic cells can be recognized by immune cells and then provoke strong inflammatory responses due to leakage of intracellular components. The current study demonstrated a substantial correlation between necroptosis and immune cells infiltrations, implying that necroptosis may facilitate the development of TAAD through activating immune infiltration and immune response.

The present study has several limitations. Firstly, although a rigorous bioinformatics analysis was conducted in the present study, our results need to be carefully assessed due to the lack of confirmation from our own experiments and clinical trials. Secondly, the sample size was relatively small, and the results of this study should be confirmed in larger prospective studies. Thirdly, immune infiltration analysis was performed based on transcriptomic data. Hence, we could not determine if necroptosis caused immune cell infiltration, or whether immune cells are also involved in the process of necroptosis. Further studies are warranted to clarify the underlying mechanisms.

## 5. Conclusions

Bioinformatics analyses revealed that the expression levels of necroptosis-related genes significantly differed between TAAD and normal aortic tissue samples. Moreover, we found the imbalance of Th17/Treg cells and the correlation between the expression of ferroptosis-related genes and the infiltration of multiple immune cell types in TAAD. The necroptosis- and immune-related factors identified in our analyses may help determine the underlying mechanisms of AD pathophysiology and identify novel targets for medical interventions.

## Data Availability

The data are public and can be downloaded from the GEO database.

## Supplementary Materials

Supplementary 1 Supplementary Materials S1: necroptosis gene set from Kyoto Encyclopedia of Genes and Genomes Pathway databases.

Supplementary 2 Supplementary Materials S2: differentially expressed genes of necroptosis in GSE153434.

Supplementary 3 Supplementary Materials S3: gene set enrichment analysis in Stanford type A aortic dissection based on GO terms.

Supplementary 4 Supplementary Materials S4: gene set enrichment analysis in Stanford type A aortic dissection based on KEGG.

Supplementary 5 Supplementary Materials S5: KEGG pathway analysis of NRDEGs in Stanford type A aortic dissection.
